# Estimated Burden of Serious Fungal Infections in Mozambique

**DOI:** 10.3390/jof4030075

**Published:** 2018-06-23

**Authors:** Jahit Sacarlal, David W. Denning

**Affiliations:** 1 Faculty of Medicine, University Eduardo Mondlane, Maputo 702, Mozambique; 2 The National Aspergillosis Centre, University Hospital of South Manchester, The University of Manchester and Manchester Academic Health Science Centre, Manchester M13 9PL, UK; david.denning@manchester.ac.uk

**Keywords:** *Pneumocystis jirovecii*, fungal infection, HIV/AIDS, epidemiology, Mozambique

## Abstract

Mozambique is a sub-Saharan African country with limited information on the burden of fungal disease. We estimated the burden of serious fungal infections for the general healthy population and for those at risk, including those infected with HIV, patients with asthma, as well as those under intensive care. We consulted the Mozambican National Institute of Statistics Population and Housing Census report to obtain denominators for different age groups. We use modelling and HIV data to estimate the burdens of *Pneumocystis jirovecii* pneumonia (PCP), cryptococcal meningitis (CM) and candidiasis. Asthma, chronic obstructive pulmonary disease and tuberculosis data were used to estimate the burden of allergic bronchopulmonary aspergillosis (ABPA) and chronic pulmonary aspergillosis (CPA). In 2016, the Mozambique population was 26.4 million with 1.8 million people reported to be HIV-infected. Estimated annual incidence of fungal infections was: 33,380 PCP, 18,640 CM and 260,025 oral and oesophageal candidiasis cases. Following pulmonary tuberculosis, estimated numbers of people having chronic pulmonary aspergillosis (prevalence) and allergic bronchopulmonary aspergillosis complicating asthma are 18,475 and 15,626, respectively. Tinea capitis is common in children with over 1.1 million probably affected. We also highlight from studies in progress of high incidences of histoplasmosis, CM and *Pneumocystis jirovecii* in adult HIV-infected patients. Prospective epidemiology studies with sensitive diagnostics are required to validate these estimates.

## 1. Introduction

Mozambique is a sub-Saharan African country, with a long Indian Ocean coastline that held its first democratic election in 1994. The country’s gross domestic product in 2016 was estimated at $11 billion, $382 per capita [1]. In 2016, 51% of the 26.4 million population were adults, of whom only 4.5% were over 60 years and life expectancy was 55 years [2]. The country has a high HIV and high tuberculosis burden [3,4]. Substantial efforts to control the HIV epidemic have resulted in significant falls in new HIV infections, and over 50% of the HIV-infected population have received antiretroviral therapy (ART) [3].

The epidemic of HIV is also a major factor that has contributed to a remarkable increase in the frequency of mucosal candidiasis; before the extensive use of highly active antiretroviral therapy (HAART) in developed countries, 80% of HIV infected patients developed oral candidiasis and about 20% developed esophageal candidiasis [5]. Many HIV-infected patients also developed cryptococcosis, *Pneumocystis* pneumonia and other lethal mycoses, such as disseminated histoplasmosis [6].

In Mozambique, there is a major lack of information on the burden of fungal disease. The emergence of new fungal diseases, some associated with HIV infection and the continuing large number of deaths in AIDS, 62,000 in 2016 [3], require improvement in laboratory diagnosis. In the National Health System (NHS), there are 314 laboratories of different levels with about 1373 employees and only 82 senior technicians [7]. Clinical microbiology, including mycology diagnosis comprising culture and antibiotic sensitivity (AST), is performed in 11 clinical laboratories at the three central hospitals, six provincial hospitals, and two general hospital laboratories. The other two provincial hospital laboratories, and some district and rural hospital laboratories are providing microbiology diagnoses based on microscopy, including gram stain. Laboratories of the Faculty of Medicine of the Eduardo Mondlane University (UEM), Health National Institute (INS) and very few private laboratories are doing mycology research studies and providing clinical diagnostics [7].

Few studies have reported on the burden of any fungal disease in Mozambique. The objective of this work is to estimate the total burden of the serious fungal diseases there, to assist in determining public health and research priorities.

## 2. Materials and Methods

Published epidemiology papers reporting fungal infection rates from Mozambique were identified. The burden of serious fungal infections was estimated for the general healthy population and for those population groups at risk, including patients living with HIV infection or AIDS (PLWHA), survivors of pulmonary tuberculosis, patients with asthma, as well as those under intensive care. We consulted the Mozambican National Institute of Statistics Population and Housing Census report to obtain denominators for different age groups [2]. Where no national data existed, we used specific populations at risk (Table 1) and fungal infection frequencies in those populations to estimate national incidence or prevalence, depending on the condition (Table 2). Data on the HIV population were extracted from the Joint United Nations Programme on HIV/AIDS (UNAIDS) 2016 Global and World Health Organization (WHO) report, and where necessary, local reports for HIV treatment and care services were used [3]. The WHO tuberculosis report was consulted to obtain data on tuberculosis patients [4]. We assumed the rate of asthma to be around 4.7% in adults [8]. Assumptions from other published reports were used to identify the most accurate denominators as explained in detail for each fungal infection below. We also assumed that no transplantation was done and COPD data was not available.

We also conducted a comprehensive literature review for published prevalence and/or incidence rates of fungal infections in Mozambique and applied these rates to the populations at risk. During the searching, abstracts were reviewed and the relevant full text articles were selected. The next step involved a manual search of the reference lists of all the selected articles to identify other relevant articles for final selection. Where no available data were found in the literature, local unpublished data was sought, and in the absence of local data, published estimates from neighboring countries were used. Due to the lack of similarity between the populations studied and used in our deterministic model published previously [9,10], conventional methods for addressing heterogeneity in systematic reviews were not applicable. Therefore, a narrative approach was taken to report the findings of the studies included. Our estimates assumed the lowest incidence rates reported and focused only on well-defined risk populations.

## 3. Results

### 3.1. Country’s Profile

In 2016, the Mozambican total population was estimated to be 26.4 million, with 51.7% females. Of the general population, 44.7% were younger than 15 years and 4.6% were above 60 years of age [2]. According to the UNAIDS 2016 report, in 2016, Mozambique had 83,000 (73,000–96,000) new HIV infections and 62,000 (50,000–73,000) AIDS-related deaths. There were 1,800,000 (1,600,000–2,100,000) people living with HIV in 2016, among whom 54% (41–63%) were accessing ART. Among pregnant women living with HIV, 80% (61–95%) were accessing treatment or prophylaxis to prevent transmission of HIV to their unborn children. An estimated number of children newly infected with HIV was 13,000 (7000–20,000) due to mother-to-child transmission in 2016 [3] (Table 1).

### 3.2. Pneumocystis jirovecii Pneumonia (PCP)

PCP is a life-threatening condition affecting patients with severe immunosuppression [12,13]. However, with early diagnosis and treatment, survival outcomes have been reported at 70% in Africa, and in well-resourced settings, may be as high as 90% [14,15].

In Mozambique, all patients with advanced immunosuppression due to HIV are recommended to receive co-trimoxazole prophylaxis for prevention of PCP. A study by Lanaspa et al. in 2015 reported a prevalence of 9.2% of PCP among children <5 years age admitted to hospital with clinical severe pneumonia diagnosed by PCR [16] (Table 2). Another study in HIV-infected patients with pulmonary infections reported 16.3% of PCP among TB patients [17,18]. PCP cases have been reported in a study done by Sacarlal et al. at Maputo Central Hospital with prevalence of 38% among adult HIV-infected patients admitted with pulmonary infections/or Kaposi sarcoma and confirmed using nested PCR [19] (Table 2).

We therefore estimated a total of 33,380 and 6624 cases of PCP in adults and children in 2016 (126 cases per 100,000 person–years and 25 cases per 100,000 person–years), respectively, assuming a 16.3% incidence in adults and 9.2% in children of those with CD4 counts under 200 × 10^6^/mL in adults and advanced HIV in children [20] (Table 2).

### 3.3. Cryptococcal Meningitis (CM)

*Cryptococcus neoformans* is the most common cause of meningitis among adults with severe immunosuppression due to HIV [21,22,23,24]. Only one study so far in Mozambique has reported 19.4% prevalence of cryptococcal antigenaemia, in severely immunosuppressed among HIV ART naïve adults hospitalized with CD4 <200 cells per µL [25] (Table 2).

We estimated the incidence of CM in 2016. By applying the 19.4% rate of cryptococcal antigenaemia, we calculated 18,640 cases of cryptococcosis (70.5 cases per 100,000 person–years) [20] (Table 3).

### 3.4. Histoplasmosis

Histoplasmosis *(H. capsulatum)* cases have been reported in a study done by Sacarlal et al. at Maputo Central Hospital with prevalence of 58% among adult HIV-infected patients admitted with clinical diagnoses of pulmonary infection/or Kaposi sarcoma and confirmed using nested PCR [19] (Table 2). Assuming this prevalence, we estimated 153 cases of histoplasmosis in 2016 at a rate of 0.58 cases per 100,000 person–years (Table 3).

### 3.5. Oesophageal Candidiasis

Another AIDS defining illness occurring among patients with advanced HIV, is oesophageal candidiasis, especially but not limited to, in those ART-naïve. Oesophageal candidiasis cases were estimated to be 75,718 at a rate of 287 cases per 100,000 person–years (Table 3), using the assumptions that 22.5% of AIDS patients not on ART and 0.5% of those on ART developed this disorder [26,27] (Table 3).

### 3.6. Oral Candidiasis

We estimated oral candidiasis to affect 184,307 Mozambicans at a rate of 698 cases per 100,000 person–years (Table 3). This burden was estimated by assuming that 90% of untreated HIV patients with CD4 counts <200 × 10^6^/mL developed oral candidiasis (Table 3).

Oral candidiasis is among the most common clinical presentations of ART naïve immunosuppressed HIV patients. About 50% of newly presenting symptomatic HIV-infected patients have oral candidiasis [28,29]. A few studies are published about estimation of oral candidiasis in Mozambican children. One is a prevalence estimation in children under 5 years admitted in a rural district hospital with severe malnutrition (6%) and with other admission diagnoses (2%) [30]. Another study shows a prevalence of 5.5% of oral manifestations in HIV/AIDS children at the DIA Pediatric Hospital in Maputo [31] (Table 2).

### 3.7. Recurrent Vulvovaginal Candidiasis

Recurrent vulvovaginal candidiasis is defined as four or more episodes per year [32]. The infection is usually caused by *Candida albicans*, less often by other species, notably *C. glabrata* which is fluconazole resistant based on information from other countries. An estimated 70–75% of women suffer from vulvovaginal candidiasis at least once in their lives, often during pregnancy [32].

Attacks of recurrent vulvovaginal candidiasis (rVVC) have been estimated to affect annually 5–8% in women between 15–50 years-old age group based on a recent systematic review by Denning et al. and from Foxman et al. [33,34,35].

There are no reports of rVVC from Mozambique, but we estimated rVVC among adult women in the general healthy population to affect 348,179 women in 2016 at a rate of 2635 woman per 100,000 person–years (Table 3). We calculated this assuming that 6% of all adult women in Mozambican have rVVC per year, unrelated to HIV infection.

### 3.8. Candidaemia and Invasive Candidiasis

Candidaemia and *Candida* peritonitis were estimated to affect 1321 and 198 patients, respectively, at rates of 5 and 0.75 cases per 100,000 person–years, respectively (Table 3). The highest incidence is most likely among patients with cancer, postsurgical patients and those on intensive care. We calculated this using the assumption that candidaemia occurs at a population rate of 5 cases per 100,000 and *Candida* peritonitis at a ratio of 1 patient with hospital-acquired (almost all postoperative) case for every two patients with candidaemia [36,37]. As blood culture is not a routine investigation in Mozambique, there are no local data to validate this estimate.

### 3.9. Other Fungal Infections

Any skin disorder affects 11% of the population according to population survey in slum areas on Beira City in Central region of the country (Table 2). Tinea capitis is one of the more common diseases [38,39]. Tinea capitis affects between 6.8% and 11.6% of primary school children in Mozambique [40,41] and 11.8% in street children in Maputo City [42] (Table 2), which is a higher rate than those in many other sub-Saharan African countries [10].

According to the national population census, there are 11,816,857 primary school aged children in Mozambique, and the country’s net primary school enrolment is 97% [2] (Table 1). We estimated from these reports that 1,181,686 school children suffer from tinea capitis each year at a rate of 4474 cases per 100,000 person–years (Table 3).

### 3.10. Allergic Bronchopulmonary Aspergillosis (ABPA)

ABPA is an occasional common complication of bronchial asthma and CF and also serves as a predisposing factor for chronic pulmonary aspergillosis (CPA) [43,44,45]. A previously described model by Denning et al. on the burden of ABPA, including one study from South Africa, reported 2.5% prevalence of ABPA among adults with asthma [46]. To estimate the burden of ABPA, we thus used published prevalence of adult asthma from a neighboring country, Malawi (4.67%), to estimate the number of adults with asthma in Mozambique [11] (Table 1).

Using the 4.67% adult asthma prevalence (682,136 adults) and the estimated 2.5% ABPA prevalence among asthmatic adults previously described by Denning et al. [47], 17,053 adults were estimated to have ABPA and in 2016 (Table 3).

Surveys of adolescents in 27 schools located in urban, suburban and semi-rural areas of Maputo showed the prevalence of asthma (13.3%) to be higher than in adults (Table 2) [48], so the ABPA rate may be an underestimate, although this disease has yet to be recognized in Mozambique.

### 3.11. Severe Asthma with Fungal Sensitization (SAFS)

We estimated the burden of SAFS in Mozambique from the adolescent asthmatic population (*n* = 218,284), rather than the total adult asthmatic population (*n* = 682,136). Severe asthma occurs in ~10% of the asthmatic population [46], and fungal sensitization is especially common in severe asthma. The prevalence of “severe attacks of asthma” has been reported between the 11.9% and 13.3% of the adolescents of 20 schools located in urban, suburban and semi-rural areas of Maputo City. Teenagers instructed in the suburban schools reported more severe asthma-like symptoms than others (*p* < 0.05) [8,48] (Table 3). We calculated from these assumptions that SAFS affects 9072 Mozambican adolescents annually at a rate of 34 cases per 100,000 person–years (Table 3). If a similar assumption was made for all adults with asthma (assuming the Malawi figure is a reasonable estimate), the number of SAFS cases would be 20,398.

### 3.12. Chronic Pulmonary Aspergillosis (CPA)

CPA occurs commonly as a sequel of several lung inflammatory conditions [45]. Treated pulmonary tuberculosis can lead to CPA as a long-term sequel [49].

In sub-Saharan Africa, the general lack of diagnosis of *Aspergillus fumigatus* causing CPA, may lead to underdiagnosis and mismanagement with most of these patients treated as cases of sputum smear-negative tuberculosis [50]. CPA presents with different radiological features, such as a simple aspergilloma, chronic cavitary pulmonary aspergillosis and chronic fibrosing pulmonary aspergillosis. Consequently, in tuberculosis endemic areas, such as Mozambique, misdiagnosis is common [49].

We calculated the incidence and prevalence of CPA using data on tuberculosis and applying the model described by Denning et al. [47]. In 2016, the total of pulmonary tuberculosis was reported in 73,470 individuals [4] (Table 1), although the estimated incidence was 159,000, 90% of which was pulmonary. According to Denning et al., the rate of CPA is 22% among the 22–35% pulmonary tuberculosis cases who develop cavities, and 2% in those without visible cavities (Table 3) [47]. Assuming that pulmonary tuberculosis is responsible for 80% of all CPA cases in Mozambique, we estimated a prevalence of 23,094 cases of CPA at a rate of 87 cases per 100,000 persons–years (Table 3) and 18,475 post TB.

### 3.13. Invasive Aspergillosis (IA)

We calculated the annual incidence of IA from severely immunosuppressed patients with leukaemia, assuming that 10% of acute myeloid leukaemia patients develop IA, and an equal number for all other leukemias [49,51]. There are no transplantation procedures done in Mozambique. We estimated 159 cases of IA in 2016 in leukaemia occurring at a rate of 0.6 cases per 100,000 person–years (Table 3). Patients with HIV probably do develop IA, and if we assume a 4% rate among those who die of AIDS, an additional 2480 IA cases are likely to be found, but not diagnosed [52]. We cannot assess how many IA cases complicate COPD (usually on admission to hospital) as COPD data are missing, and likewise, how many cases complicate lung cancer therapy is unclear, as this diagnosis is usually not made and only 363 cases are registered [53].

## 4. Discussion

Mozambique lost people infected with AIDS with estimated numbers of 34,000 in 2015 and 62,000 in 2016, respectively. Cryptococcal disease and PCP appear to be common and histoplasmosis has also been reported; oesophageal candidiasis is also common and contributes to morbidity and weight loss in these already vulnerable patients. There are no useful data on aspergillosis and other rarer infections; the newly described disseminated *Emergomyces* infections from South Africa may be present, but we have no data to date. The estimated numbers of PCP cases at >33,000 in adults and >6000 in children per year greatly exceed prior estimates from Mozambique and dwarfs other sub-Saharan estimates, and is based on data generated in the country. The estimate for cryptococcal disease and meningitis cases (*n* = 18,640) comes from Rajasingham et al., using CrAg prevalence of 6.6%. In fact, this estimate may be a significant underestimate based on 19.4% prevalence now documented [20], and would push the estimate to 54,790. Given that the average of death of patients with CM is 35 years (and younger for PCP), these deaths are a devastating loss to Mozambique.

Pulmonary TB is a major problem, too, with a 36% mortality and over 140,000 cases with 91,500 survivors annually. Given this, there is no surprise that we estimate 5860 new cases of chronic pulmonary aspergillosis cases complicating TB each year, which translates into 5-year prevalence of 18,475 cases, assuming a 15% annual mortality. Given that 45% of cases of WHO notified TB in Mozambique are smear negative (clinically diagnosed) and that Oladele et al. in Nigeria [9] found a rate of CPA of 10% and 19% in HIV negative and HIV positive smear negative survivors, our estimates might be slightly high, but the cross-sectional nature of Oladele’s study design omits those who already died of aspergillosis and does not include CT scanning which is superior to chest X ray. In addition, some patients had negative *Aspergillus* IgG serology in Nigeria with overt radiological changes, such as fungal balls or positive serology with radiological findings but no symptoms [52]. However, there is clearly a significant need to address CPA in the TB and respiratory population in Mozambique with *Aspergillus* serology and offer appropriate therapy.

In 2006/2007, 10% of children with the estimated number of 1,181,686 had tinea capitis. This is, therefore, a significant public health problem, which requires addressing, as many of these children (2–10%) will have kerion (painful inflammation of the scalp), and some will have permanent hair loss.

The major challenge in Mozambique for diagnosing fungal diseases is the low number of clinical laboratories with capacity and ability to performed mycology testing. Only 11 clinical laboratories at the three central hospitals, six provincial hospitals, and two general hospital laboratories do culture and AST, usually performing this test with some difficulties. Some laboratories, such as the Faculty of Medicine of the UEM, INS and a few private laboratories, can do the mycology diagnoses when involved in a research project. The more important challenges include a lack of human resources, weak technical competencies among microbiology laboratory staff, inconsistent provision of supplies (consumables and reagents), a lack of basic equipment and equipment maintenance programs, deficient laboratory information systems and poor infra-structural layout and biosafety. Testing algorithms are not yet standardized, resulting in significant differences in methods among laboratories.

The Faculty of Medicine of the UEM and other laboratories and the Ministry of Health (MoH) are making big efforts to secure access to better quality microbiological testing and more effective support to clinicians. This will help their clinical decision-making and provide more reliable support to public health professionals with responsibility for formulating policies related to the control and management of infectious diseases on hospitals, including fungal diseases. However, investments in personnel, include training, assessment and appropriate guidance, as well as the introduction of effective recruitment and retention practices, together with ensuring that technical competence is valued, attractive and protected, are all needed in the country.

The other issue is availability of antifungal drugs in the NHS. Only amphotericin B is available for CM but flucytosine is not, like the rest of sub-Saharan Africa. Miconazole, fluconazole and itraconazole are rarely available.

## 5. Conclusions

Approximately 1,834,947 (6.9%) people in Mozambique are estimated to suffer from a serious fungal infection each year. Tinea capitis in children and recurrent *Candida* vulvovaginitis are the most common, with HIV fungal complications being the next most common. We also highlighted from studies in progress, the very high incidence of histoplasmosis, CM and PCP in adult HIV-infected patients. Prospective epidemiology studies are required to validate these estimates.

## Figures and Tables

**Table 1:** Country’s profile. Population and rates required to calculate burden of serious fungal infections (2017).

Population at Risk	Population	Reference
Demographic data	Total population—26,423,623	[2]
	Total of children (<15 years)—11,816,857	
	Total number of adults—14,606,766	
	% women over 60 = 4.5	
HIV/AIDS	Estimated number of people living with HIV in 2016 = 1,800,000 (1,600,000–2,100,000)	[3]
	New HIV infections in 2016 = 83,000 (73,000–96,000)	
	Proportion of diagnosed cases on ARVs = 54% (41–63%)	
	Estimated HIV prevalence among adults 15–49 Years =10.5%	
	Estimates AIDS-related deaths = 62,000 (50,000–73,000)	
	Estimated AIDS-related deaths among adults 15+ Years = 34,000	
	Estimated number of adults living with HIV receiving antiretroviral treatment (ART) = 802,659	
Tuberculosis	Tuberculosis case notifications in 2016 (total new cases) = 73,470	[4]
	Incidence (include HIV+ TB only) = 159,000 (103,000–227,000)	
	Rate (per 100,000 population) = 551 (356–787)	
	Extra pulmonary tuberculosis cases 2016 = 7347	
	Pulmonary tuberculosis cases in 2016 = 32,329	
	51% of TB patients who were tested were HIV-positive (2015)	
Asthma	Prevalence of asthma in adolescents = 13.3%	[8]
	Prevalence of asthma in adults (taken from Malawi) = 4.67%	[11]

ART, antiretroviral therapy.

**Table 2:** Prevalence and incidence in previous reports used to estimate the burden of serious fungal infections.

Disease	Population	Prevalence	Reference
**Oral candidiasis**	Children under the age of 5 years admitted to a rural district hospital with severe malnutrition	6.0%	[30]
	Children under the age of 5 years admitted to a rural district hospital with other admission diagnosis	2.0%	[30]
	HIV+/AIDS patients at the DIA Pediatric Hospital of Maputo	5.5%	[31]
**Tinea capitis**	Children in two rural primary schools	6.8–11.6%	[40]
	In streetchildren of Maputo	11.8%	[42]
	Among native children	3.6%	[39]
	Children from suburban primary school	9.6%	[41]
**Skin Disorders**	Population survey in slum areas on Beira	11.0%	[38]
***Pneumocystis jirovecii* pneumonia**	HIV-infected patients with pulmonary infections	16.3%	[18]
	Children <5 years age admitted to hospital with clinical severe pneumonia (confirmed by nested PCR)	9.2%	[16]
	Adult HIV-infected patient admitted on HCM with pulmonary infections and/or Sarcoma Kaposi (confirmed by nested PCR)	38.0%	[19]
**Histoplasmosis**	Adult HIV-infected patient admitted on HCM with pulmonary infections and/or Kaposi sarcoma (confirmed by nested PCR)	58.0%	[19]
**Asthma**	Adolescents asthmatics of 27 schools located in urban, suburban and semi-rural areas of Maputo	13.3%	[8]
	Adolescents asthmatics of 20 schools located in urban, suburban and semi-rural areas of Maputo	11.9%	[48]
**Cryptococcal meningitis**	Cryptococcal antigenaemia among HIV ART naïve adults hospitalized with CD4 <200 cells per µL	19.4%	[25]

**Table 3:** Burden of fungal diseases in Mozambique according the main risk factors (2016).

Population: 26,423,623 (2016)	Number of Infections per Underlying Disorder per Year					Estimated Number of Cases	Rate per 100,000	Predominant Groups at Risk
	None	HIV/AIDS	Respiratory	Cancer	ICU			
*Pneumocystis jirovecii* pneumonia (adults)	-	33,380	-	-	-	33,380	126.3	AIDS
*Pneumocystis jirovecii* pneumonia (child)	-	6624	-	-	-	6624	25.1	AIDS
Cryptococcal meningitis	-	18,640	-	-	-	18,640	70.5	AIDS
Oesophageal candidiasis	-	75,718	-	-	-	75,718	286.6	HIV/AIDS
Oral Candidiasis		184,307				184,307	697.5	HIV/AIDS
Candidaemia	-	-	-	925	396	1321	5.0	Immunocompromised patients
*Candida* peritonitis	-	-	-		198	198	0.75	Immunocompromised patients
Recurrent vaginal candidiasis (4×/year or more)	348,179	-	-	-	-	348,179	2635 *	Adult women
Allergy bronchopulmonary aspergillosis (ABPA)	-	-	17,053	-	-	17,053	64.54	Asthma patient
Severe asthma with fungal sensitization (SAFS)	-	-	9072	-	-	9072	34.3	Asthma patient
Chronic pulmonary aspergillosis	-	-	18,475	-	-	18,475	69.9	Tuberculosis patient ^&^
Invasive aspergillosis	-	-	-	159	-	159	0.6	Hematological malignancy
Mucormycosis	-	-	-	53	-	53	0.2	Hematological malignancy
Histoplamosis	-	153	-	-	-	153	0.6	AIDS/Respiratory disease
Tinea capitis	1,181,686	-	-	-	-	1,181,686	4472	Poor Hygiene
Total burden estimated	1,529,865	260,178	59,346	1137	594	1,836,374		

ICU, Intensive care unit; * rate for females only; ^&^ assumes 80% of cases are related to TB, 20% other pulmonary conditions.
